# NADase as a target molecule of in vivo suppression of the toxicity in the invasive M-1 group A Streptococcal isolates

**DOI:** 10.1186/1471-2180-10-144

**Published:** 2010-05-17

**Authors:** Ichiro Tatsuno, Masanori Isaka, Masaaki Minami, Tadao Hasegawa

**Affiliations:** 1 Department of Bacteriology, Nagoya City University Graduate School of Medical Sciences, 1 Kawasumi Mizuho-cho Mizuho-ku, Nagoya 467-8601, Japan

## Abstract

**Background:**

NAD-glycohydrolase (NADase) secreted by M-1 group A streptococcal (GAS) isolates are suspected as one of the virulence factors to cause severe invasive disease including streptococcal toxic shock-like syndrome (STSS). M-1 GAS strains were divided into three groups based on NADase activity: high activity, low activity and no activity in our previous report.

**Results:**

The representative high activity isolates taken from STSS patients showed higher virulence compared with isolates from the low activity group, when used to infect mice. The knockout mutant of the *nga *gene, which encodes NADase also showed reduced virulence in a mouse infection study. The cloned *nga *gene was able to significantly complement the lost virulence. In addition, the solution containing purified recombinant IFS, which is an inhibitor of NADase, partially rescued mice infected with *S. pyogenes*.

**Conclusions:**

These results indicate that NADase is important for the virulence of *S. pyogenes *in vivo and is the potential target to suppress the virulence.

## Background

Group A streptococcus (GAS) is a gram-positive bacterium that infects the upper respiratory tract, including the tonsils and pharynx, and is responsible for post-infectious diseases such as rheumatic fever and glomerulonephritis. In addition, GAS causes severe invasive disease including necrotizing fasciitis [1-6].

Although the mechanism of severe invasive disease is still unknown, NAD-glycohydrolase (NADase) secreted by GAS is suspected as one of the virulence factors [7]. NADase has the ability to cleave β-NAD^+^, which is universally important in numerous essential redox and energy-producing biological reactions, depleting intracellular NAD pools [8,9]. NADase is also toxic for bacterial cells themselves, therefore, GAS encodes *ifs *gene whose product (IFS) is an endogenous inhibitor of NADase activity and localized in the bacterial cytoplasmic compartment [9,10]. NADase precursor exists as an inactive complex with IFS [9,10]. In vitro, intoxication of keratinocytes with NADase was associated with cytotoxic effects [11,12]. Bricker *et al*. presented that NADase enhances GAS virulence in vivo using mouse models [13]. These results enabled us to further study the NADase as a target molecule to reduce GAS virulence. However, another study of GAS infection among aboriginal people in Australia found no relationship between NADase production and severity or outcome of GAS infection [14]. Furthermore, we recently reported that M-1 group A streptococcal isolates were divided into three groups based on NADase activity: high activity, low activity and no activity [15], whereas we did not find that low and high levels of the NADase activity correlated with severity of GAS human infection (data not shown). Meanwhile, Ajdic *et al*. reported that among 73 strains isolated from patients with mostly invasive GAS infections from a recent outbreak of streptococcal infection, 67 (92%) were NADase producer [16], although strains isolated from patients with non-invasive GAS infections were not assayed. It is unknown why the 8% strains isolated from patients with mostly invasive GAS infections were not NADase producer. Therefore, we thought that before taking up the study of our interest, it should be further determined how NADase is important as a virulence factor for severe invasive disease. We mainly focused on the following two points: (i) How do NADase activity levels correlate with virulence? (ii) If NADase is important for severe invasive disease, and whether it is possible that IFS suppresses the severity. In this study, we present further evidences to prove the importance of NADase in severe invasive disease.

## Methods

### Bacterial strains

Streptococcal strains were isolated as causative organisms from invasive diseases patients in Japan (Table 1). *S. pyogenes *(GAS) strain SF370, which is prevalent as the database reference isolate (accession NC_002737), was provided by the courtesy of J. J. Ferretti [17,18]. Streptococcal strains were cultured in brain heart infusion (E-MC62, EIKEN Chemical Co., Tokyo, Japan) supplemented with 0.3% yeast extract (BD, Sparks, MD, USA) (BHI-Y) broth unless otherwise described.

**Table 1:** M-1GAS clinical isolates used in this study

Isolates	place^#^	Isolated year
SF370	America	1985
1529	Japan (Chiba)	1990-2000
KN01	Japan (Aichi)	1990-2000
MDYK	Japan (Aichi)	2000 ~
MUY	Japan (Mie)	2000 ~
GT01	Japan (Gunma)	2000 ~
FI01	Japan (Fukushima)	2000 ~
CR01	Japan (Aichi)	2000 ~
IYAT	Japan (Fukushima)	2000 ~

All isolates, except for SF370, are derived from invasive diseases.

# Japanese Cities were described in parenthesis.

### Quantitation of NADase activity in bacterial supernatant

NADase activity was determined by the method of Stevens *et al*. [19] as described previously [15].

### Construction of the recombinant His-IFS and His-TarC proteins

The *ifs *gene of pGST-Nga_GT01 _(IFS) [15] was amplified by PCR with *Extaq *DNA polymerase (Takara Bio, Ohtsu, Japan) using primers IFS-F (BamHI) (5'-AGGAAGTAAC**GGATCC**TATAAGGTGC-3') and IFS-R (5'-ATGTGTCAGAGGTTTTCACCG-3'). Oligonucleotide IFS-F(BamHI) contained a restriction site for *Bam*HI (shown in bold in the primer sequence). The amplification product, which contained a restriction site for *Sal*I, was digested with *Bam*HI and *Sal*I, and cloned into pQE-80L (Qiagen, Hilden, Germany) to yield pHis-IFS, whose insert was sequenced.

Plasmid pHis-TarC encoding a His-tagged carboxyl terminal domain of an *Escherichia coli *aspartate chemoreceptor (named as His-TarC) was constructed by subcloning a 1.1 kb *Kpn*I fragment of pIT6 [20] into pQE-80L.

### Purification of the recombinant His-tagged proteins

The His-tagged IFS fusion protein was induced and purified under native conditions as described in the manufacture's protocol (Qiagen), with the following modification. To induce the His-IFS fusion protein, 1 mM IPTG was added to a logarithmic-phase culture of *E. coli *JM109/pHis-IFS and shaken for 3 h at 37°C. A total of 100 ml of the liquid culture was transferred to a centrifuge tube and centrifuged to sediment the cells. The pellet was resuspended in 10 ml ice cold PBS + 1% Triton X-100. After a freeze (-80°C)/thaw and a sonication at 170 W for 2 min (Insonator 201M, Kubota, Tokyo, Japan), insoluble material was removed by spinning it at full speed (16 000 *g*) for 10 min. One ml of the 50% Ni-NTA slurry was washed twice with 4 ml of Milli-Q water, equilibrated with 1 ml of PBS + 1% Triton X-100, added to the 10 ml cleared lysate and mixed gently by rotating at room temperature for 20 min. The lysate-Ni-NTA mixture was loaded into a column and washed three times with 4 ml wash buffer. The protein was eluted with PBS + 250 mM Imidazole. The protein was verified using SDS-PAGE and anti-RGS-His antibody (Qiagen) or by dose-dependent inhibition of NADase activity of both GAS culture and the GST-Nga fusion protein constructed in a previous report [15].

The His-TarC was induced and purified by the same method described above. In addition, characterization by SDS-PAGE confirmed that the IPTG-dependently induced recombinant protein was purified as essentially a single band of the expected size (31 k Dalton) (data not shown).

### Mouse model of invasive skin tissue infection

All animal studies have complied with federal and institutional guidelines. The ability of *S. pyogenes *to cause local skin lesions and necrosis in mice after skin inoculation was assessed using a procedure similar to that described elsewhere [21]. In brief, 3-week-old female ICR mice (10-12 g) were anesthetized by ketamine-xylazine injection, and the hair was cut from the left flank using scissors and/or electric shaver to bare the skin, unless otherwise indicated. Bacteria (0.1 ml; 1 × 10^7 ^cfu per mouse) grown in BHI-Y were injected with a 27-gauge needle just under the surface of the skin so that a superficial bleb was raised immediately below the skin surface. The number of colony-forming units injected was verified for each experiment by plating bacteria on BHI-Y or sheep blood agar plates (with or without kanamycin) and counting colony-forming units.

The purified recombinant His-IFS or His-TarC was injected as follows: (1) on day 0, 25 μg (per 0.1 ml) was inoculated together with bacteria in the left flank. It was confirmed that both His-IFS and His-TarC had no effect on bacterial viability and growth (data not shown), and (2) on days 2-4, 50 μg (per day) was inoculated intraperitoneally. The bacterial viability (and growth) was assessed by incubating the remaining mixture of bacteria and either His-IFS or His-TarC used on the day 0 for 1 to 6 hours, and counting colony-forming units on BHI-Y or sheep blood agar plates. Because it is difficult to increase injection volume in the skin, we decided to increase the concentration of IFS per ml of injection solution. Preliminary test showed highest concentration (no dilution) was more effective at reducing GAS virulence than any of the IFS dilutions tested (data not shown). Thus, we used the highest concentration to add as much IFS as our possible.

### Creation of *nga *mutant of strain GT01

*Escherichia coli *JM109 was used to propagate plasmid constructions. Non-polar inactivated mutant of *nga *was constructed via double-crossover allelic replacement in the chromosome of *S. pyogenes *GT01. To construct the plasmid for the *nga *knockout mutant, the 5' end of *nga *(fragment 1) was amplified with oligonucleotide primers ngaGT-n1 (5'-G**GCTAGC**GAACAGATGTGAAGGTTCTG-3') with an *Nhe*I restriction site and ngaGT-c1 (5'-TCC**CCCGGG**TTTCTCATGTAAACCACCT-3') with an *Sma*I restriction site, and the 3' end of *nga *(fragment 2) was amplified with ngaGT-n2 (5'-TCC**CCCGGG**ATAGGAAGTAACAATATGT-3') with an *Sma*I restriction site and ngaGT-c2 (5'-GG**ACTAGT**ATGTTAGCTTTCAATTGGGT-3') with an *Spe*I restriction site. Oligonucleotides ngaGT-n1, ngaGT-c1, ngaGT-n2 and ngaGT-c2 contained a restriction site for *Nhe*I, *Sma*I, *Sma*I and *Spe*I, respectively, (shown in bold in the primer sequence). Fragment 2 was digested with *Sma*I and *Spe*I for insertion into multi-cloning site 2 of the pFW12 plasmid [22]. The resulting plasmid was then digested with *Nhe*I and *Sma*I, and both the spc2 DNA fragment containing *aad9 *(promoterless spectinomycin resistant gene), which was obtained from a *Sma*I digested fragment of pSL60-2 [23], and the *Nhe*I-*Sma*I-digested fragment 1 were inserted. This plasmid, *nga*::*aad9*/pFW12, was a suicide vector for *S. pyogenes*. For the preparation of competent cells, strain GT01 was harvested at early- to mid-log phase (OD_660 _= 0.4 to 0.5) and washed twice with 0.5 M sucrose buffer. The constructed suicide vector *nga::aad9*/pFW12 was transformed into strain GT01 by electroporation. The conditions of electroporation were 1.25 kV/mm, 25 μF capacitance and 200 Ω resistance, using Gene Pulser II (Bio-Rad, Hercules, CA, USA). After incubation at 37°C for 3 h, competent cells were spread onto BHI agar plates containing 0.3% yeast extract and spectinomycin (final concentration 100 μg/ml). Selected colonies on the plates were cultured. Cultured bacteria were washed once with saline, resuspended in 10 mM Tris, 1 mM EDTA and boiled for 10 min. Genomic DNA was obtained from the supernatant of boiled bacteria. The double-crossover replacement was analyzed using genomic DNA by PCR and successful double-crossover replacement was further confirmed by DNA sequencing.

### Cloning of *nga *gene

All PCR reactions for plasmid construction were undertaken as previously described [15].

The *nga*_*GT01 *_of *S. pyogenes *strain GT01 was amplified by PCR with *Extaq *DNA polymerase using primers nga-n4Eco (5'-G**GAATTC**ATGAGAAACAAAAAAGTAAC-3') and sloC2 (5'-ATCATCCGTTTTCTGACCTG-3') and cloned into pGEM-T easy (Promega, Madison, WI, USA) to yield pNGIe1, whose insert was sequenced. Oligonucleotide nga-n4Eco contained a restriction site for *Eco*RI endonuclease (shown in bold in the primer sequence). The *nga*_*GT01 *_gene is oriented in the opposite direction as the *lacUV5 *promoter. An *Eco*RI fragment containing the *nga*_*GT01 *_gene of pNGIe1 was sub-cloned into pLZ12-Km2 [24] to yield pLZN2, whose insert was sequenced for verification.

To construct pLZN-RBS, inverse PCR with Pyrobest DNA polymerase (Takara) using the primers LZ-R0 (5'-CCGTCGAC**CTCGAG**GGGGGGC-3') and nga-RBS1 (5'-CCG**CTCGAG**ATATAAGGTGGTTTAC*ATG*AGAAACAAAAAAGTAAC-3') was performed to add a potential ribosome-binding site (16 bp) to *nga *encoded on pLZN2. Oligonucleotides nga-RBS1 and LZ-R0 contained a restriction site for *Xho*I endonuclease, the potential ribosome binding site and/or start codon for the *nga *gene, respectively (shown in bold, underline and italic in the primer sequence, respectively). The amplification product was digested with *Xho*I and self-ligated. The insert was sequenced for verification.

To construct pLZN-RBSII2, inverse PCR with PrimeSTAR™ HS DNA polymerase (Takara) using the primers nga-RBS2 (5'-CCGGGGCCCTTAAAAATAATATAAGGTGGTTTAC*ATG*AG-3') and LZ-R3 (5'-CTCGAGGGGGGGCCCATCAGTC-3') was performed to add the further upstream DNA sequence (10 bp) to the potential ribosome-binding site encoded on pLZN-RBS. A oligonucleotide nga-RBS2 contained the upstream DNA sequence, the potential ribosome binding site and start codon for the *nga *gene (shown in dotted underline, underline and italic in the primer sequence, respectively). The amplification product was self-ligated and the insert was sequenced for verification.

### Statistical analysis

The significant difference of virulence (mortality) between low and high NADase activity groups was ascertained as follows. The mortality of mice infected with each GAS isolate, but not mean mortalities produced by pooling multiple isolates into the two groups, was determined. The four mortalities in the low NADase activity group and the four mortalities in the high NADase activity group were compared using an unpaired *t *test http://www.graphpad.com/quickcalcs/ttest1.cfm.

Survival times were assessed using a log-rank comparison. R software was used for statistical analysis http://bioinf.wehi.edu.au/software/russell/logrank/. *P *value ≤ 0.05 was considered significant.

## Results

### Correlation of NADase activity levels and virulence

**Figure 1:**
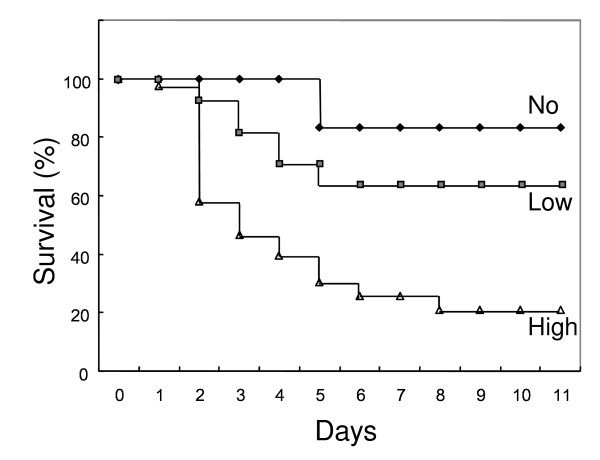
**Survival after skin inoculation with GAS isolates with different NADase activities**. The survival times of 28 and 43 mice infected with GAS isolates belonging to low- and high-activity groups in Table 2, respectively, were shown. In addition, the survival time of mice infected with strain SF370 belonging to the no-activity group was also shown as a reference, since it is possible that the old strain differs considerably in genetic background compared with the other more contemporary eight isolates of Table 2 as described previously [7].

The levels of detectable NADase activity produced by clinical isolates of M-1 GAS were divided into two groups (low-activity and high-activity) in our previous study [15]. It is possible that isolates belonging to the high-activity group are more virulent, possibly causing invasive infection at higher severity and/or with lower dose. To investigate this possibility, we used a mouse model for the invasive soft-tissue infection, which is currently the most accepted available method for this type of in vivo experiment. As shown in Table 2, after skin inoculation with M-1 GAS isolates belonging to the high-activity group, 80%, 60%, 100% and 67% of the mice were dead within a week, respectively, whereas with the isolates belonging to the low-activity group, 29%, 33%, 67% and 17% of the mice died, respectively (*P *= 0.0272 for unpaired *t *test). The survival curves (Figure 1), based on the data of Table 2 showed that no mouse died after day 8 on the study.

**Table 2:** Virulence (Mortality) to mouse of GAS isolates with different NADase activity

NADase	Isolate	Mortality^a ^(Death/Trial)	NADase^b^
Low activity	1529 KN01 MDYK MUY	29% (2/7) 33% (3/9) 67% (4/6) 17% (1/6)	3.37 ± 0.66 6.19 ± 0.52 2.95 ± 0.26 2.97 ± 0.95

High activity	GT01 FI01 CR01 IYAT	80% (12/15) 60% (6/10) 100% (12/12) 67% (4/6)	57.03 ± 3.65 59.40 ± 4.76 114.30 ± 8.67 87.25 ± 5.22

No activity^c^	GT01Δnga SF370	0% (0/8) 17% (1/6)	0.49 ± 0.13 -0.44 ± 0.80

a, Mortality was determined on Day 11.

b, NADase activity (units) ± standard error are indicated. One unit of NADase activity is defined as the amount (μg) of β-NAD cleaved per hour per μl culture supernatant as described previously [15].

c, Strain SF370, which encodes an inactive form of Nga [15] was added as negative control.

### Mouse infection model using *nga *knockout mutant and complemented strain

To investigate the extent with which NADase contributes to GAS virulence in the mouse model, *nga *gene encoding NADase of strain GT01 was replaced with an antibiotics marker. The resulting GT01*Δnga *did not show any detectable NADase activity and mortality in the invasive soft-tissue mouse-infection test (Table 2). Therefore, we tried to complement the phenotype using a plasmid pLZN2 in which only the coding region of *nga *is cloned. However, the complementation study using GT01Δ*nga *(pLZN2) strain was not successful in restoring survival times (Table 3). Unsuccessful complementation might be due to insufficient NADase activity in the GT01Δ*nga *(pLZN2) strain (NADase activity: 1.28 ± 0.12 U). Therefore, two additional plasmids (pLZN-RBS and pLZN-RBSII2) were constructed containing 16 and 26 base pair upstream DNA sequences encoding the potential ribosome-binding site, which is lacking in pLZN2 respectively (see Materials and Methods in detail). The resultant GT01Δ*nga *(pLZN-RBSII2), but not GT01Δ*nga *(pLZN-RBS), strain enhanced virulence compared to the mutant in the mouse model (*P *= 0.019 for comparison of survival times). The result of the GT01Δ*nga *(pLZN-RBS) strain may also be due to the same reason that the strain was non-functional, since it contained only slightly improved levels of NADase activity (1.78 ± 0.03 U).

**Table 3:** Virulence (Mortality) to mouse of GT01Δnga with or without cloned *nga *gene

Strain	**Mortality**^**a**^	**NADase**^**b**^
GT01 (pLZ12-Km2, vector)	73% (8/11)	14.12 ± 1.30
GT01Δnga (pLZ12-Km2, vector)	0% (0/17)	0.04 ± 0.06
GT01Δnga (pLZN2, *nga*)	0% (0/11)	1.28 ± 0.12
GT01Δnga (pLZN-RBS, *nga*)	0% (0/10)	1.78 ± 0.03
GT01Δnga (pLZN-RBSII2, *nga*)	29% (4/14)	4.57 ± 0.17

Bacteria were cultured in BHI-Y broth supplemented with kanamycin (100 μg/ml).

a, Mice were observed for 8 days, because no mouse died after day 8 on previous study (see Figure 1).

b, NADase activity was determined as described in Table 2.

Furthermore those results encouraged us to construct plasmids containing longer upstream DNA sequences than what is present in pLZN-RBS and pLZN-RBSII2. However these plasmids were not successfully constructed (data not shown, see Discussion in detail).

### Assessment of body weight change in mouse infection model experiment

**Figure 2:**
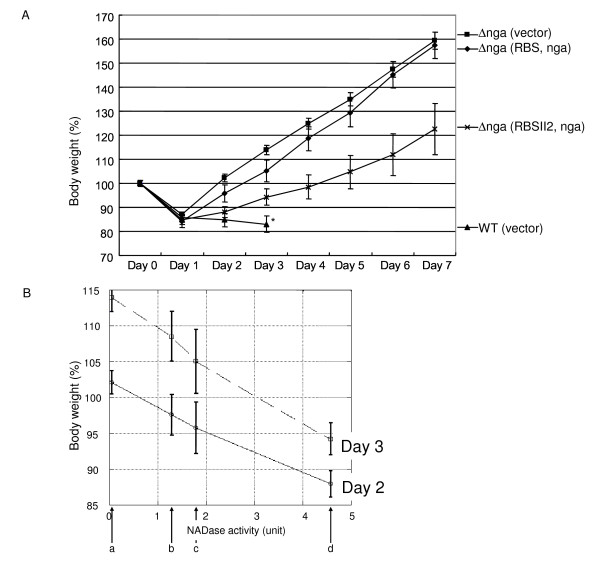
**Virulence (based on body weight change) to mouse of GT01*Δnga *with or without cloned *nga *gene**. (A) The change in body weight (% of the first weight) post-infection was shown in a week (* as a reference, the parental strain was shown in three days, because most mice died within this period). (B) Relationship of body weight and NADase activity was shown on days 2 and 3. NADase activities (0.04, 1.28, 1.78 and 4.57 U, respectively) of (a) GT01Δnga (pLZ12-km2), (b) GT01Δnga (pLZN2), (c) GT01Δnga (pLZN-RBS) and (d) GT01Δnga (pLZN-RBSII2) was plotted on the horizontal axis, respectively (see Table 3 or the text for NADase activity of each strain). The gradual increase in body weight (%) depended on higher NADase activity of the strains. The error bars indicate the standard error of the means.

First, we judged the virulence based only on the mortality rate. Although GT01*Δnga *(pLZN2) and GT01*Δnga *(pLZN-RBS) did not kill the injected mice (Table 3), possibly due to insufficient NADase activity, we found that there were some mice which exhibited a poor health condition but eventually survived. Hence, we also evaluated virulence of GAS infection in mice by monitoring body weight. In this method, lower body weight implies a more severe form of disease. We hypothesized that the body weight of mice injected with the complement should be lower than those injected with GT01*Δnga *(pLZ12-Km2: control vector). Our results indicated that the average rates of increasing body weight in mice injected with the complement strain GT01*Δnga *(pLZN2) were lower than in mice injected with GT01*Δnga *(pLZ12-Km2), but this difference was not statistically significant (data not shown). GT01*Δnga *(pLZN-RBS) injection in the mouse model showed slightly lower increasing rates of body weight than did GT01*Δnga *(pLZN2) (data not shown) and GT01*Δnga *(pLZ12-Km2: control vector) (Figure 2A). In addition, when the body weight on days 2 and 3 post-infection was analyzed, GT01*Δnga *(pLZN-RBSII2) with the highest NADase activity showed the slowest increasing rate of body weight (Figure 2B).

### IFS-inhibition of the virulence of the GAS strain GT01

**Figure 3:**
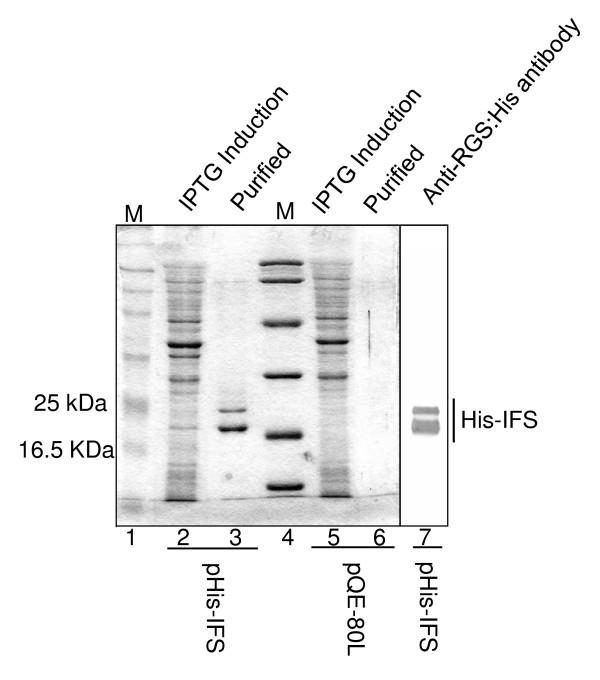
**Purification of His-tagged IFS protein**. The protein overexpressed with IPTG in *E. coli *JM109 having pHis-IFS (lane 2), but not in *E. coli *JM109 having the control vector pQE80L (lanes 5 and 6), was purified as shown in lane 3. The protein was detected by anti-RGS:His antibody to confirm the expected His-tagged product (lane 7).

**Figure 4:**
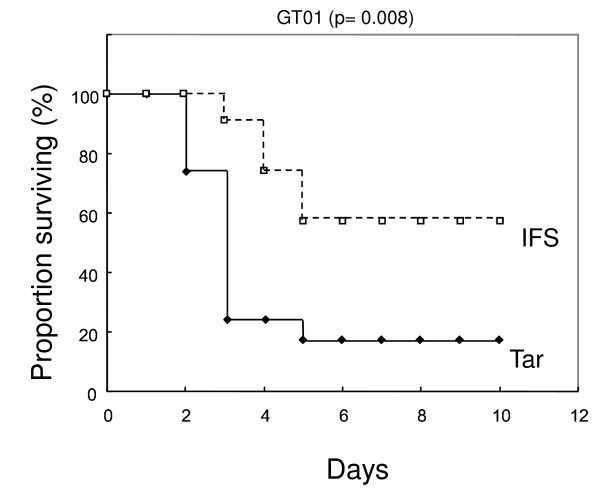
**Inhibition of the mortality in mouse of a GAS GT01 clinical isolate by His-IFS**. The solution including His-IFS reduced the virulence of the GAS to 42% mortality (5 death/12 trial) compared with 83% (10/12) of the control His-TarC (*P *= 0.008 for comparison of survival times).

If purified IFS is able to suppress GAS virulence in the mouse-infection model, it would support the role of NADase in vivo. For this experiment, His-IFS was purified (Figure 3) and used in the mouse-model infection. Meanwhile, as an unrelated protein, His-TarC which is a His-tagged carboxyl terminal domain of an *E. coli *aspartate receptor was used. As shown in Figure 4, the solution containing purified His-IFS, but not the control His-TarC, significantly reduced the virulence of GAS. The control protein was not effective for GAS virulence (Figure 4) because the mortality and the survival times did not decrease and prolong, respectively, compared with the result of GT01 infection without treatment (see GT01 strain in Table 2 for comparing the mortalities, data not shown for survival times).

## Discussion

Recent reports have suggested that NADase is important for enabling GAS to cause invasive disease [13,19]. These reports created our interest in NADase as a target molecule to reduce the GAS virulence. However, before studying the ability of a NADase-inhibitor to reduce GAS virulence, we felt NADase itself should be further characterized in its virulence causing role based on the following two reasons. (i) In M type-3 clinical isolate used in the previous study, the difference between mortality of mice infected with the *nga *strain and the parental strain was about only 25% [13]. Meanwhile, we recently reported that M-1 group A streptococcal isolates are divided into three groups based on NADase activity: high activity, low activity and no activity [15]. If a high-activity isolate is used to measure mortality of mice infected with GAS compared with the *nga *strain, the difference could be wider than if a low activity isolate were used. Indeed, in this study, we found that the difference between mortalities of mice infected with GT01 (12/15 = 80% death), a high-activity isolate, and the GT01*Δnga *(0/8 = 0% death) was 80% (see Table 2). In addition, the difference in mice mortality between the cases of GT01 (pLZ12-Km2, vector plasmid) and GT01*Δnga *(pLZ12-Km2) was 73% (see Table 3). This result shows that the GT01 isolate could provide an advantage compared with the M type-3 clinical isolate when studying the ability of a NADase-inhibitor to reduce GAS virulence, which is our original interest. (ii) To our knowledge, the reduced virulence of the *nga*-deletion mutant of GAS has never been successfully complemented using a cloned *nga *gene. It is common knowledge that complementation tests in vivo are not easily accomplished due to increased technical problems when compared to an in vitro study. In such cases, some alternative methods can be used. For example, Bricker *et al*. [13] constructed two independent *nga*-deficient mutants and showed that they have similar phenotypes. In this study, we added two more points of supportive data. We showed that an *nga *knockout GAS strain possessing a cloned *nga *gene partially restored virulence (Figure 2 and Table 3). In addition, we showed that a solution containing purified IFS suppressed the virulence of GAS in the experimental mouse-infection model (see later for additional discussion).

Although the data of Table 2 support our conclusion described earlier, some of the individual data were inconsistent with each other. For example, some of the strains belonging to low- and high-activity groups showed similar survival curves. However, this is not surprising because multiple factors play a role in GAS virulence, and the productions of virulent factors differ among the strains [25]. Therefore, it is important to compare groups including multiple, but not single, strains.

Regarding Nga knockout mutant and complementation, the NADase activity for the GT01 strain carrying the pLZ12-Km2 vector was different from that of GT01 alone (reported in Tables 2 and 3). Indeed, the virulence between the two strains also appears to be slightly different from each other, although we were unable to explain the reason.

Although the plasmid pLZN-RBSII2 conferred significant virulence to the *nga *strain when compared to a control vector (Table 3 and Figure 2), we found that the strain *nga *(pLZN-RBSII2) produced only 8% of the NADase activity found in the wild type strain. In order to restore NADase levels to near normal, we attempted to construct plasmids containing longer upstream DNA sequences than what is present in pLZN-RBS and pLZN-RBSII2. However these plasmids were not successfully constructed, possibly due to the potential toxicity of over produced NADase to bacterial cell.

As shown in Figure 4, injection of NADase inhibitor (His-IFS) significantly rescued mice from strains GT01. To further investigate the potential of the His-IFS solution, we tested strain CR01, which showed the highest virulence in the mouse-infection model among our collected strains (see Table 2). Although His-IFS alone was not sufficient to significantly rescue mice from the strain CR01, a combination of His-IFS solution and ampicillin was able to significantly decrease GAS virulence in mice compared with ampicillin alone (unpublished data). These results also show that NADase activity occurs in vivo and can be inhibited.

Using western blot analysis, we detected two bands from pHis-IFS using anti-RGS-HIS antibody (Figure 3). Based on the specificity of this antibody, we attributed the smaller band to degradation of the His-IFS protein.

The higher virulence of strain CR01 when compared to the other isolates belonging to high activity group (Table 2) may not only be due to higher level of NADase activity, but also due to additional unknown factors. For example, two-dimensional gel electrophoresis demonstrates that CR01 presents a different pattern of secreted extracellular proteins compared to the other isolates belonging to high activity group, including markedly lower level of the SpeB protein (unpublished results). Further analysis of the strain CR01, although the less representative strain among the high activity isolates had not been focused on very much in this study, would be a very interesting advance for the field.

Finally, we should discuss the discrepancy between NADase activity being important to the virulence of *S. pyogenes *during in vivo mouse models and our epidemiological data showing that low and high levels of NADase activity do not correlate with the severity of the *S. pyogenes *isolates in human infection. One possibility is that there is no statistical difference due to low sample number which is a result of a very small number of cases of the STSS disease. There is another possibility. After human passage, the isolated *S. pyogenes *could be different from the original strain which caused the infection due to getting genetic mutations. It is difficult to directly confirm the virulence of the passed isolates, since humans are the only natural hosts for the organism. When dealing with organisms, which lack a non-human natural host, we can never be perfectly certain and therefore must rely on additional accumulated supportive (usually indirect) evidence. If our purified His-IFS (NADase inhibitor) is able to rescue STSS patients in future that could provide a more ethically acceptable form of direct evidence.

## Conclusions

We have presented further supportive evidence that NADase is important for severe invasive disease of *S. pyogenes *in vivo using the experimental mouse model. Furthermore, we provided useful evidence that the NADase is the potential target to suppress the virulence.

## Abbreviations

GAS: group A streptococci; IFS: immunity factor for *Streptococcus pyogenes*; NADase; NADase (also Nga): NAD-glycohydrolase.

## Authors' contributions

IT conceived the study. IT and TH designed and performed the experimental work with help by MI and MM. All authors contributed to analyze data. IT wrote the original manuscript. TH helped to craft the final manuscript. All authors approved the final manuscript.
